# Process optimization and mechanism study of acid red G degradation by electro-Fenton­Feox process as an in situ generation of H_2_O_2_

**DOI:** 10.3906/kim-2002-10

**Published:** 2021-02-17

**Authors:** Hailong SUN, Yingwu YAO*, Feng WEI, Qiang ZHAO, Baichen LIU, Liman ZHANG

**Affiliations:** 1 Hebei University of Technology, School of Chemical Engineering and Technology, Tianjin P.R. China

**Keywords:** Electro-Fenton, dyeing wastewater, acid red G, kinetics

## Abstract

Dye-contaminated wastewaters are industrial wastewaters that are difficult to treat using traditional biochemical and physicochemical methods. In the present work, the acid red G was removed as a model pollutant by the electro-Fenton process for the first time. The anode and cathode used by the electro-Fenton process were iron plate and graphite felt, respectively. It was concluded that under the optimal conditions of current density = 20 mA cm^-2^, pH = 3 and initial Na_2_SO_4_ concentration = 0.2 M, the removal rate of acid red G (ARG) with an initial concentration of 300 mg L^-1^ could reach 94.05% after 80 min of electrolysis. This reveals that the electro-Fenton-Feox process used in this work has an excellent removal efficiency on acid red G. The required reagents (Fe^2+^ and H_2_O_2_) were generated by the electrode reaction, while the optimal generation conditions and mechanism of •OH, H_2_O_2_, and Fe^2+^ were investigated. By testing •OH, H_2_O_2_, and Fe^2+^ agents at different pH and current densities, it was revealed that the electro-Fenton reaction was most efficient when the current density was 20 mA cm^-2^, and the pH was 3. Moreover, the removal rate of ARG is consistent with first-order reaction kinetics.

## 1. Introduction

The dyeing process is a key procedure in the textile industry that reflects the color, functionality and feel of the fabric and plays an essential role in the quality of the final product. On the other hand, the dyeing process also involves a large consumption of water. With the rapid development of the dyeing industry, its emissions account for a growth inthe proportion of industrial wastewater discharge, and it has become one of the key pollution sources in the industry [1]. Due to the type and structural complexity of the dye itself, its biodegradability can be weak, and the application of conventional biological treatment techniques is challenging [2].

Azo dyes are one of the most widely used synthetic dyes in the textile and garment-dyeing industry. They are used for dyeing and printing various natural and synthetic fibers, as well as being the coloring agents of paints, plastics, and rubber. Azo dyes are mainly characterized by the presence of at least one azo group (-N = N-). Furthermore, thereduction or cleavage of azo dye bonds produces different reaction intermediates,which can cause carcinogenesis [3]. Therefore, it is crucial to treat the azo dyes before they can enter the environment and natural water systems. In order to achieve this, different physicochemical methods (such as adsorption, stripping, and precipitation) are used to remove synthetic dyes from wastewater generated by the textile industry. On the other hand, conventional methods of treating these synthetic dye-polluted wastewaters have relatively low efficiency, and often they cannot eliminate the synthetic dye from the wastewater or the method can produce secondary wastewater containing reaction intermediates which can be even more harmful [4]. Therefore, recent studies on non-biodegradable substances have focused on destructive methods rather than non-destructive methods [5].

Electrochemical treatment is one of the most exciting and efficient methods to treat the organic pollutantsmentioned above [6]. In particular, advanced electrochemical oxidation processes (EAOP) have been extensively studied to achieve promising results for the efficient removal of organic dyes. The electrochemical advanced oxidation processes have the advantages of high safety, strong oxidation ability of pollutants,and low toxicity[7]. The main principle of EAOP is that the hydroxyl (•OH) produced in the electrochemical process is the active and most aggressive oxygen-based species that can react with almost all types of biomolecular, organic, or inorganic substances [8]. It has a very strong oxidizing electrode potential (2.8 V) [9], which is the most active inorganic oxidant except fluorine and its oxidizing ability far exceeds that of ordinary chemical oxidants. As an EAOP, electro-Fenton (EF) is a widely used method to degrade high-concentration organic pollutants in wastewaters[10]. It generates Fe^2+^ and H_2_O_2_ directly by electrolysis, and then reacts to produce •OH to attack the organic pollutants (Fe^2+^ + H_2_O_2_ → •OH + OH^-^ + Fe^3+^)[11]. Compared with the traditional Fenton reagent oxidation methods, this method can avoid the increased cost of H_2_O_2_ and the potential hazards in storage and transportation, to maintain a continuous concentration of H_2_O_2_ and can regenerate Fe^2+^ more efficiently [12]. Therefore, in recent years, investigations regarding its applicabilityhave received special attention. It has been successfully used to degrade various types of persistent organic pollutants, such as dyes, phenols, and halogenated hydrocarbons [13]. 

Acid red G(ARG) is an azo dye. In this work, ARG was used as a model pollutant, being removed by EF-Feox process with an iron plate as the anode and graphite felt as thecathode for the first time. Moreover, all the Fenton reagents were generated by electrochemical reactions at the anode and cathode. Fe^2+^ could be obtained by oxidizing the iron plate through the electrical pathway, and H_2_O_2_ was produced by the reaction of the reduction of the O_2_ in the cathode [14]. The main reactions on the electrodes and solutions were as follows [9]:

Cathode:

(1)O2+2H++2e-→H2O2

(2)2e-→H2

Anode:

(3)Fe~2e-→Fe24

(4)2H2O→O2+4H++4e-

The Fe^2+^ in the system can react with H_2_O_2_ produced in the reaction formula (1), (eq. (4)-(7)):

(5)Fe2++H2O2→ •OH+OH-+Fe3+

(6)Fe3++H2O2→Fe2++•O2H+H+

(7)Fe3++•O2H→Fe2++O2+H+

(8)Fe3++e-→Fe2+

The formed •OH reacts quickly with organics, causing its oxidation:

(9)Organic pollutants + • OH → oxidation intermediates

(10)Intermediates + • OH →CO2+H2O + inorganic ions

Taking into account the aspects mentioned above, the main aim of our investigations was tofind the optimal experimental parameters. Based onthe optimized process conditions of the EF-Feox method, this experiment investigated the generation of •OH, Fe^2+^, and H_2_O_2_ under different current density and pH.

## 2.Experimental

### 2.1. Chemicals

All chemicals were analytical grade and were used without further purification. ARG purchased from Shanghai Macklin Biochemical Co. Ltd. Table 1 summarizes its physicochemical properties. The solutions required for all experiments were prepared using deionized water as the solvent.

**Table 1:** Characteristics of ARG.

English name	ACID RED 1
Molecular	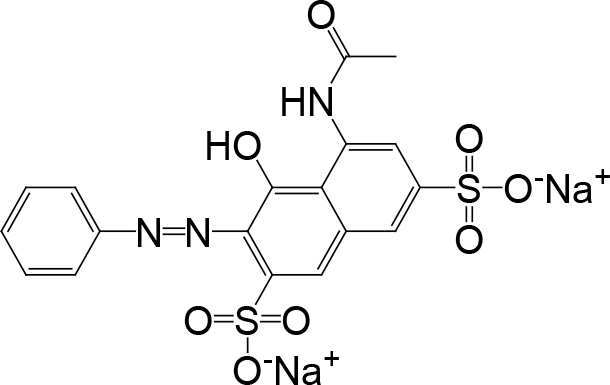
Molecular formula	C_18_H_13_N_3_Na_2_O_8_S_2_
CAS no.	3734-67-6
Molecular weight, g/mol	509.42

### 2.2. Experimental procedure

Electrochemical oxidation experiments were performed in a 250-mL beaker. Iron plates (30 cm^2^ in area) were used as the anode, while graphite felt of the same size was used as a cathode. First,200 mL of ARG solution was added to the reactor, while NaOH and H2SO4 solutions were used to adjust the pH of the wastewater to the desired value. The anode and cathode were placed vertically and parallel to each other, and the actual plate area was determined to be 15 cm^2^. A digital DC power supply (MP1560D, China) with a digital display was used as the current source in all the experiments. The operating parameters discussed and optimized in this study include current density, initial pH, initial concentration of ARG, the dosage of Na_2_SO_4_, and plate spacing. In these experiments, we filled the air to the cathode at a rate of 2 L/min. The concentration polarization caused by theformation of Fe^2+^ and H_2_O_2_ in the solution was controlled by stirring.The generation of •OH, Fe^2+^, and H_2_O_2_ at different current densities and pH was determined in the first instance without adding ARG. In order to accurately measure the actual production of H_2_O_2_, the anode material was replaced with platinum and the amount of generated H,2O_2_ was measured in an environment where the Fenton reaction did not occur.

### 2.3. Analytical methods

The changes in the concentration of ARG were determined by measuring the absorbance at a fixed wavelength (505 nm) [15], and the corresponding absorption maximum was measured using the INESA L5 UV-Vis spectrophotometer (Shanghai, China). The following formula was used to calculate the removal rate of the solution:

Removal rate =((A_0_-A )/A_0_) × 100%

whereA0 is the absorbance of the solution before the reaction of the dye and A is the absorbance of the dye solution after different times oftreatment [16].

Determination of the concentration of Fe^2+^ was carried out using 1,10-phenanthroline spectrophotometry [17], Fe^2+^ ​​can form a stable orange-red complex with 1,10-phenanthroline, this complex has the largest absorbance at 510 nm. Different concentrations of complexes have different absorbance at 510 nm according to Lambert-Beer law, and the concentration of Fe^2+^ can be obtained by measuring the absorbance according to the colorimetric principle.

Determination of H_2_O_2_ was performed using potassium titanium (IV) oxalate spectrophotometry [18]. After adding potassium titanium oxalate to the sample, an orange-yellow complex was formed immediately. After the complex was stabilized, the absorbance at 385 nm was measured. The concentration of H_2_O_2_ was calculated according to Lambert-Beer law and colorimetric principle.

•OH was measured by the fluorescence method, and coumarin was used as the capping agent of OH. When •OH attacked coumarin, it produced 7-hydroxycoumarin (7HC) with stable fluorescence characteristics (Coumarin + •OH →7HC) [19]. The concentration of coumarin was 2 mol/L and was determined to be the optimal concentration for detection. Using F97PRO fluorescence spectrophotometer (Lengguang, China), 7HC was estimated by measuring the fluorescence intensity at 456 nm. The amount of •OH produced can be indirectly obtained by measuring the concentration of 7HC. It should be emphasized that the concentration of •OH cannot be measured directly using chemical probes such as coumarin, but rather the amount of •OH produced or the cumulative concentration of products of the reaction with •OH can be estimated [14].

## 3. Results and discussion

### 3.1. Degradation of ARG by EF-Feox process and comparison with other methods

**Figure 1:**
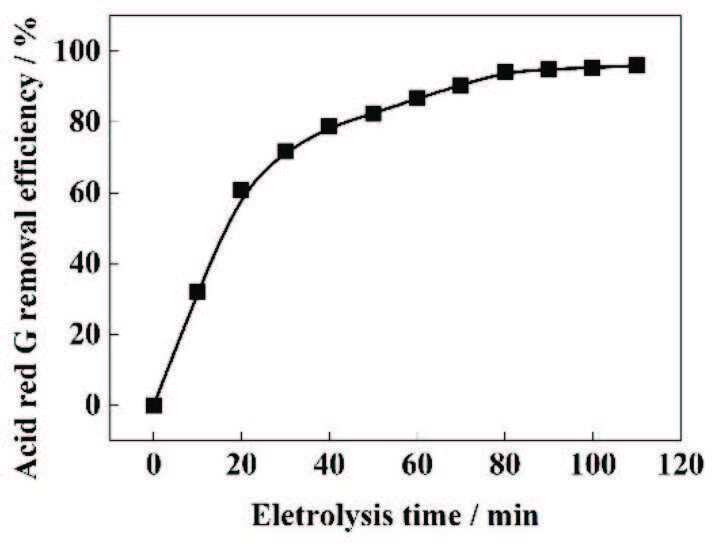
Time dependence of removal efficiency.

Generally speaking, the removal efficiency of pollutants will increase as the reaction time increases;however, too long a reaction time will also cause energy loss. It can be seen from Figure 1 that as the reaction proceeded, the removal rate of ARG gradually increased.In the first 20 min, the curve rose clearly, indicating that the reaction proceeded faster, and the removal rate of ARG was higher, reaching 60.78%.Beyond 20 min, a slow oxidation phase was observed by monitoring dye degradation, when the reaction time proceeded to 80 min, the removal rate of ARG reached 94.05%.In the next 30 min, the removal rate of ARG increased only by 1.86% to 95.91%. During the initial stage of the reaction, Fe^2+^ and H_2_O_2_were rapidly generated, and their concentration continuously increased, which accelerated the progress of the EF reaction. As the reaction time increased, the concentration of ARG gradually decreased, while the concentration of intermediate products and side reaction products increased. These side reactions and intermediates caused a gradual decrease in the rate of global reaction [20]. 

The current degradation methods for ARG are mainly photocatalysis and adsorption technologies (as shown in Table 2), and they have shown good removal effects.However, compared with the EF-Feox method used in this research, the dye concentration that the above two technologies can handle is lower, and the reaction time is very long, so the degradation efficiency is low.In addition, the degradation efficiency of the photocatalyst and adsorbent is reduced after repeated use. Lei et al. [25] used the pulse electrochemical oxidation method to degrade the solution with high ARG concentration and showed a good degradation effect, but the degradation time was relatively long and the energy consumption was large.

**Table 2:** Currently known ARG degradation methods.

Degradationmethod	Materials (catalyst, adsorbent,electrode material)	Initial concentrationof dye (mg/L)	Reaction time (min)	Removalefficiency (%)	References
Photocatalytic	KNb_3_O_8_	60	120	85.5%	[15]
Photocatalytic	Ag / AgCl / K_6_Nb_10.8_O_30_	50	100	100%	[21]
Adsorption	Fe_3_O_4_/MIL-101(Cr)	50	120	97.9%	[22]
Adsorption	Polypyrrole nano-fibers with hierarchical structure	200	90	Around 80%	[23]
Adsorption	Magnesium-aluminum-layered double hydroxides (Mg-Al-LDHs)	100	150	Around 90%	[24]
Pulsed electrochemical	Anode:Ti/Sb-SnO_2_/α-PbO_2_/β-PbO_2_ electrode Cathode: two Ti mesh with the same size	200	120	100%	[25]

Experimental conditions: initial dye concentration = 300 mg/L, current density = 20 mA/cm^2^, pH = 3, Na_2_SO_4_ = 0.2 M, and plate spacing = 2 cm

### 3.2. Effect of current density

**Figure 2:**
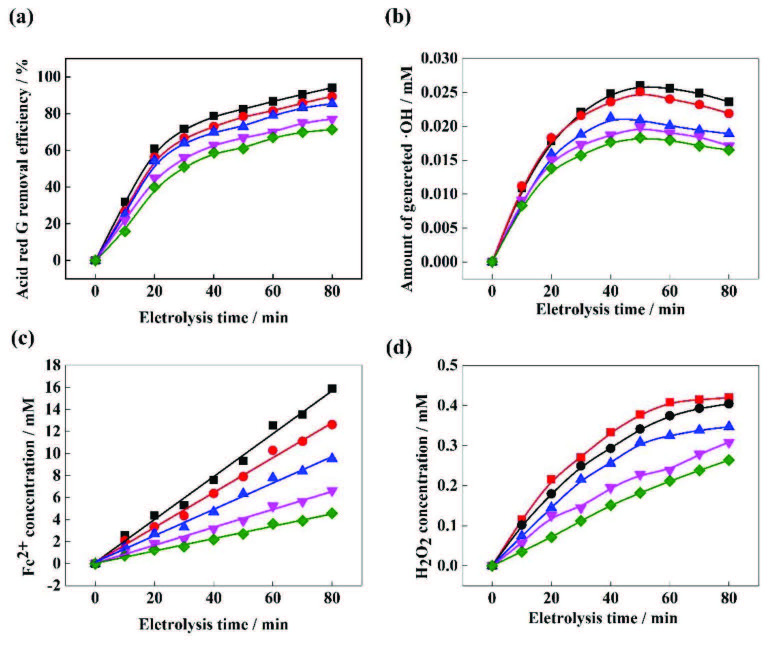
(a) The influence of current density on the removal ratio of the ARG. (b) The influence of current density on the generation of •OH. (c) The influence of current density on the elution of Fe^2+^. (d) The influence of current density on the production of H_2_O_2_. Experimental conditions: initial dye concentration =300 mg/L, pH = 3, Na_2_SO_4_= 0.2 M, and plate spacing = 2 cm. (◆) 5 mA/cm^2^, (▼)10 mA/cm^2^, (■) 15 mA/cm^2^, (■) 20 mA/cm^2^, (●) 25 mA/cm^2^.

Under the conditions of initial dye concentration of 300 mg/L, pH of 3, electrolyte concentration of 0.2 M, and plate spacing of 2 cm, the current density was adjusted to study the effect on the degradation efficiency of ARG and the yield of •OH, Fe^2+^, and H_2_O_2_. As shown in Figure 2a, after the 80 min of degradation, the removal rate of the ARG solution increased first with the current density, from 71.27% at J = 5 mA/cm^2^ to 94.05% at J = 20 mA/cm^2^. When the current density was further increased, the removal rate dropped to 89.33% at J = 25 mA/cm^2^.

The change in the degradation efficiency of ARG was due to the change in the amount of •OH produced (reaction (9) and (10)). The amount of as-generated•OH at different current densities was investigated (Figure 2b). The increase in •OH production corresponded to the cumulative concentration of 7HC of •OH attackingcoumarin [17]. The decrease in the production of •OH could be partially attributed to the degradation offormed 7HC by •OH. At the initial stage of the formation of •OH, the agent only reacted with coumarin because of the relatively high concentrations of coumarin in the solution.After a specific reaction time, the concentrations of coumarin and 7HC became comparable, and •OH began to react not only with coumarin, but also with 7HC.Therefore, as the formation rate of 7HC increased, the degradation of 7HC caused by •OH increased [26].

As can be seen from Figure 2b, under different current densities, the amount of generated •OH in the initial stage increased with the increase of current density and reached a peak around 40-50 min. After 80 min, the amount of generated •OH reached 0.0165, 0.0171, 0.0189, 0.0219, and 0.0236 mM when the current density was 5, 10, 15, 25, and 20 mA/cm^2^, respectively. It can be seen that the optimal generating current density of •OH is around 20 mA/cm^2^. When J≤ 20 mA/cm^2^, the yield of •OH increased with the further increase of the current density,this is because the increase in current density promoted the formation of Fe^2+^ and H_2_O_2_ (Figures 2c and 2d), which in turn promoted the Fenton reaction. As the current density increased, the electrochemical solubility of sacrificial iron anode and the amount of generated H_2_O_2_ increased (reaction (3)) [27] and the electrochemical regeneration of Fe^2+^ (reaction (8)) at the cathode wasenhanced with the increase of current density. 

However, the production of •OH at J= 25 mA/cm^2^ waslower than that at J= 20 mA/cm^2^.The decrease in •OH production may be due to the suppression of the electric production of Fe^2+^ and H_2_O_2_ at the electrode.It can be seen in Figure 2c that the rate of Fe^2+^ formation began to decrease when the current density was 25 mA/cm^2^. This is because the side reaction (reaction (4)) that produced O_2_occurred at the anode and hindered the dissolution of Fe^2+^ at a higher current density. Figure 2d shows that the increase in H_2_O_2_ content was not significant (3.6%) at a current density of 25 mA/cm^2^, this phenomenon can be attributed to the corresponding increase in current density. As the current density increased, the side reaction of hydrogen evolution (reaction (2)) and the decomposition of H_2_O_2_ at the anode became more and more important. Moreover, the aggravation of the side reaction of electrolysis generated a large amount of Joule heat, resulting in a decrease in current efficiency [28]. Therefore, when the current density increased from 20 mA/cm^2^ to 25 mA/cm^2^, the cumulative concentration of H_2_O_2_ increasedslightly.

### 3.3. Effect of the initial pH 

**Figure 3:**
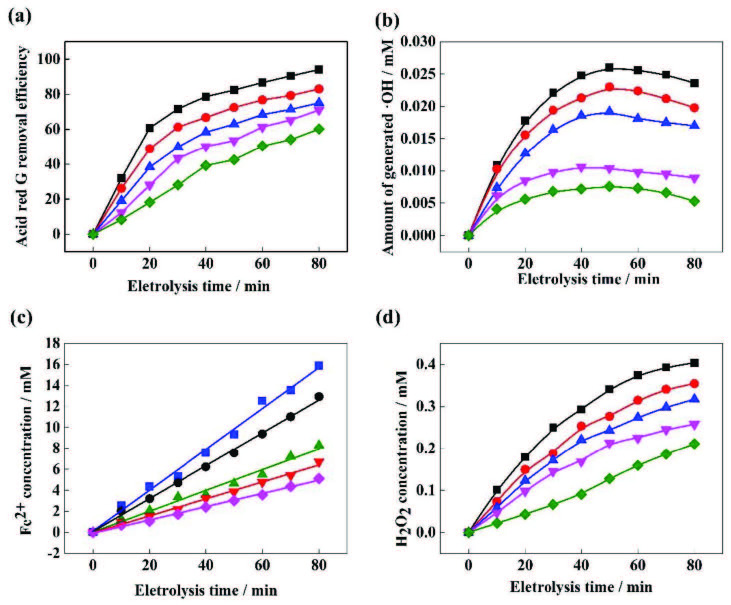
(a) The influence of pH on the removal ratio of ARG. (b) The influence of pH on the generation of •OH. (c) The influence of pH on the elution of Fe^2+^. (d) The influence of pH on the elution of H_2_O_2_. Experimental conditions: initial dye concentration = 300 mg/L, current density = 20 mA/cm^2^, Na_2_SO_4_ = 0.2 M, and plate spacing = 2 cm. (◆) pH = 1, (¸) pH = 2, (■) pH = 3, (●) pH = 4, (▼) pH = 5.

Under the conditions of the current density of 20 mA /cm^2^, electrolyte concentration of 0.2 M, and electrode spacing of 2 cm, the pH of the reaction was changed and its effect on ARG degradation efficiency and •OH, Fe^2+^, and H_2_O_2_ production was examined. As shown in Figure3a, the removal rate of the ARG solution increased with the increase of pH within the range of pH ≤ 3 after 80min reaction: from 60.12% of pH 1 to 94.05% of pH 3. After that, the removal rate of ARG gradually decreased with a further increase in pH.In the high pH environment, the oxidation ability of •OH will decrease [2]. In addition, pH is an important factor that affects the concentration of Fe^2+^ and H_2_O_2_ and therefore affects the process of the Fenton reaction and the generation of •OH.

It can be seen in Figure 3b that the generation of •OH in the EF-Feox processwas significantly affected by the pH of the solution. Initially,the amount of generated •OH quickly increased and then gradually decreased after the amount of generated •OH reached the maximum value. The yield of •OH obtained at pH 1, 2, 3, 4, and 5 was 0.0053, 0.017,0.0236, 0.0198, and 0.0089 mM after 80 min, respectively. These results confirmed that the optimum pH for the generation of •OH in the EF-Feox Process was pH 3. If the pH value was higher, the amount of produced •OH species was significantly reduced,because more and more Fe^2+^ formed Fe(OH)_3_ through the side reaction under high pH conditions(4Fe^2+^ + 10H_2_O + O_2_ → 4Fe(OH)_3_ + 8H^+^), which profoundly reduced the efficiency of the EF reaction [2], so the free Fe^2+^ concentration decreased. It can be seen in Figure 3c that when pH = 4 and 5, the amount of Fe^2+^ produced decreased to 6.71 and 5.13 mM after 80 min, respectively. As shown in Figure 3d, increasing the pH inhibited the production of H_2_O_2_. When the pH > 3, the active point for producing hydrogen peroxide decreased[6], with a side reaction occurring in the system (2H_2_O_2_ → 2H_2_O + O_2_), which could reduce the content of H_2_O_2_ in the solution [29].

As it is shown in Figure 3b, the production of •OH was greatly inhibited at pH 1 and 2 because when pH < 3, the H_2_O_2_ produced in situ formed a stable oxonium ion (H_2_O_2_ + H^+^ → H_3_O_2_+) and, as a result, the generation of •OH by the Fenton reaction was sloweddown [30]. In addition, the free Fe^2+^ concentration in the solution decreased when the pH of the solution was too low (pH1), this is due to the formation of Fe(H_2_O)^2+^ [31]. Moreover, Fe^3+^ formed Fe(III)-hydroxyl complexes (Fe(OH)^+^) in an acidic environment, the formation of this complex hindered the regeneration of Fe^2+^ (reaction (6)) [32]. Therefore, the reduction of ARG removal efficiency was also observed in strong acidic solution (pH < 3).

### 3.4. Effect of initial concentration

**Figure 4:**
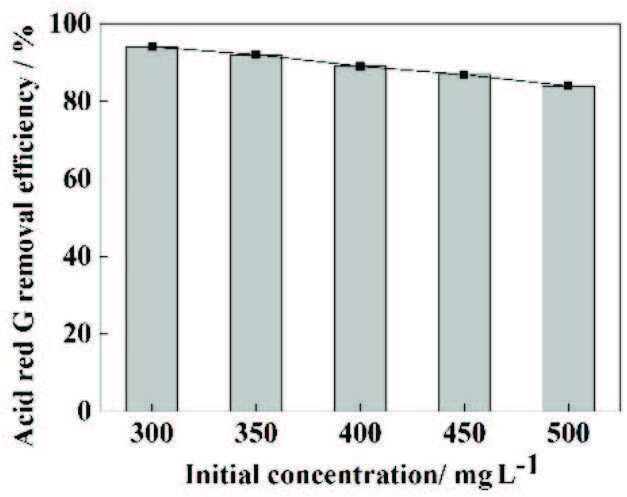
The influence of initial concentration on the removal ratio of ARG. Experimental conditions: pH = 3, current density = 20 mA/cm^2^, Na_2_SO_4_ = 0.2 M, and plate spacing = 2 cm.

In the “real field”, the actual concentration of sewage can continuously change, so it is essential to explore the initial concentration of pollutantsand their effect on the efficacy of removal methods [33]. Five different dye concentrations (300, 350, 400, 450, and 500 mg/L) were selected to study the effect of initial dye concentration on the degradation of ARG.As shown in Figure 4, as the initial concentration of the dye increased, the removal rate of ARG gradually diminished. When the initial concentration was 300 mg/L, the removal rate of ARG reached 94.05% in 80 min. The reasons for the above phenomenon could be described as follows: after determining the operating conditions, only a certain amount of •OH can be generated, so only a certain number of organic molecules can be processed [34]. When the dye concentration was low, the dye molecules could fully react with •OH, and the removal rate was high. The generated •OH was not enough to degrade it when the dye concentration was high; therefore, the degradation efficiency was reduced.

### 3.5. Effect of supporting electrolyte

**Figure 5:**
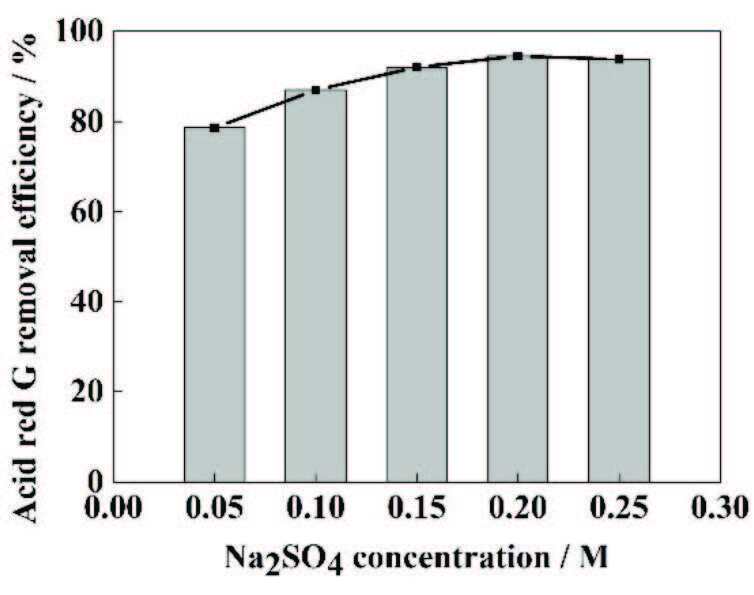
The influence of supporting electrolyte on the removal efficiencies of ARG. Experimental conditions: initial dye concentration = 300 mg/L, pH = 3, current density = 20 mA/cm^2^, and plate spacing = 2 cm.

Due to the poor conductivity of the ARG solution by itself, it is necessary to add some electrolytes to improve the conductivity of the solution in order to achieve better removal efficiency. Contreras et al. [17] compared different electrolytes to determine that Na_2_SO_4_ was the best performing electrolyte. Therefore, in the present work Na_2_SO_4_ wasused as an auxiliary electrolyte. Figure 5 shows the efficiency of different Na_2_SO_4_ concentrations on the removal of ARG during the EF-Feox process. Mainly, with the increase of Na_2_SO_4_ concentration, the removal rate of ARG increased. However, when the electrolyte concentration exceeded 0.2 M, the ARG removal rate began to decrease. As the electrolyte concentration increases, the concentration of free ions in the solution may also increase, and the possibility of particle collisions in the reaction system also increases. At the same time, under constant currents, higher conductivity could reduce the voltage in the reactor and therefore increaseenergy consumption [35]. As the dosage of Na_2_SO_4_ is increased further, the removal rate of ARG is slightly reduced. This phenomenon is due to the consumption of •OH by SO_4_^2-^ (SO_4_^2-^ + •OH + H^+^ → SO_4_^-•^ + H_2_O) [36]. The increase of the salt content also increases the viscosity and density of the solution and thus inhibits the mass transfer process in the solution [20] so that the dye molecules cannot fully contact with the •OH, which inhibits the removal of dye. In summary, the optimum concentration of Na_2_SO_4_ is 0.2mM.

### 3.6. Effect of the electrode gap

**Figure 6:**
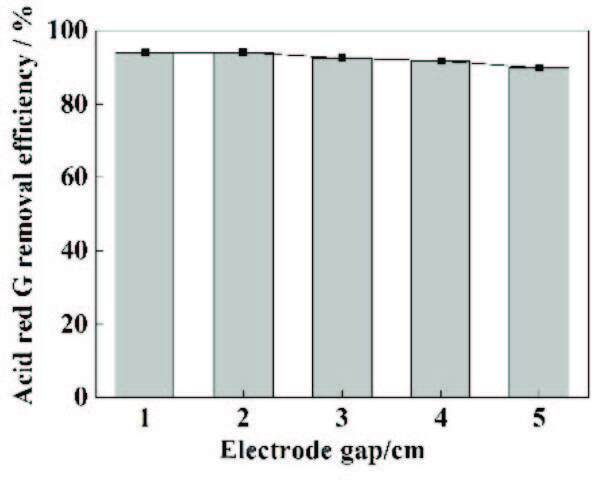
The influence of the electrode gap on the removal ratio of ARG. Experimental conditions: initial dye concentration = 300 mg/L, pH = 3, current density = 20 mA/cm^2^, and Na_2_SO_4_ = 0.2 M.

The effect of electrode spacing on the treatment efficiencies was investigated. The experimental results are shown in Figure 6. When the electrode spacing was 2 cm, the ARG removal rate was the highest. When the electrode spacingwas increased further, the as-obtained removal rates gradually decreased. The electrode spacing was determined by the magnitude of the electric field strength in the cell and the potential difference between the liquid phase and the anode [16]. When the spacing between the plates was 1 cm, the anode and cathode were too close to each other, so the •OH and other strong oxidizing intermediate products produced by Fenton reagent were directly reduced at the cathode, without enough time to oxidize the targeted organic pollutants [37]. At the same time, if the distance between plates was too small, the bubbles generated during electrolysis could cause the fluctuation of water samples, making the distribution of pollutants uneven, affecting the stability of the treatment efficiency, which may cause the breakdown of the setup, and could cause damage to the electrode material and circuit [38]. With the increase of plate spacing, the reduction of •OH mentioned above was proved to lessen until it could be ignored entirely. However, at the same current intensity, with the increase of plate spacing, the tank voltage was also increased energy consumption, and the side reactions could occur and increase, such as hydrogen and oxygen evolution (reaction (2), (4))[39]. Therefore, the electrode spacing was chosen to be 2 cm.

### 3.7. UV-VIS spectrum change of solution during ARG degradation

**Figure 7:**
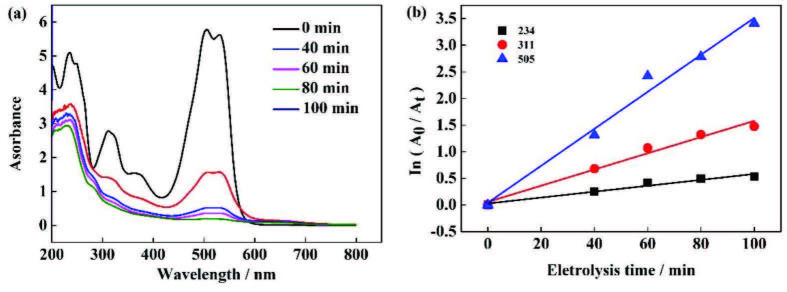
(a) UV-visible spectroscopy of ARG during the degradation of Electric Fenton. (b) Change in absorbance/ concentrations at three characteristic wavelengths.

The ultraviolet spectra of ARG, measured by UV-Vis spectrophotometer can be observed in Figure 7a, where the characteristic absorption peak of ARG in the visible region was in the range of 490 to 560 nm (attributable to azo bond, n→π*). The characteristic absorption peak in the ultraviolet region had two regions: 200 to 280 nm (attributed to the benzene ring, π→π*) and 300 to 380 nm (attributed to the naphthalene ring, π→π*). As the electrolysis progressed in time, the absorbance values ​​in the ultraviolet region and the visible regioncontinuously decreased, indicating that the ARG molecules in the solution were continuously decomposed due to the action of electrolysis, and the gradual destruction of corresponding azo bond, benzene ring structure, and naphthalene ring structure.

Figure 7b shows the relationship between the trend of the absorbance values ​​at the three different characteristic wavelengths shown in Figure 7a, by first-order reaction. From the graph, the rate constant could be obtained as follows: 0.0347 min^-1^ (505 nm, R^2^= 0. 9789), 0.0152 min^-1^ (311 nm, R^2^ = 0. 9746) and 0.0055 min^-1^ (234 nm, R^2^= 0.9533). It can be observed that the absorbance at 504 nm decreased the fastest, followed by that at 311 nm and 234 nm, indicating that the azo bond was most easily broken during the electrochemical degradation of ARG. In the actual process, the color of the ARG solution became lighter until it was colorless, visible also with the naked eye. The bond-breaking rate of the benzene ring structure was lower than that of the naphthalene ring structure, which may be due to the formation of the intermediate structuresfrom the benzene ring, right after the naphthalene ring structure was broken. The final products of Fenton system’ s theoretical degradation of azo dyes are CO_2_ and H2O, but in actual situations, in order to save energy, the reaction time cannot be extended indefinitely, so the dye cannot be fully mineralized. A small amount of benzoic acid, phenol, and their derivatives are usually present in the final stage of •OH oxidation of dyes, as well as a series of carboxylic acids [40 42]. With a further extension of the reaction time, these intermediate products will be mineralized into CO_2_ and H_2_O (reaction (9) and (10)). Murrieta et al. [43] reported that ARG produces maleic acid and oxalic acid in the process of degradation using the Fenton system. It is expected that maleic acid is formed by oxidation and cleavage of azo dye aromatic rings, while oxalic acid is the final by-product of degradation of long chain aliphatic carboxylic acid. In the presence of iron ions, these acids exist mainly as Fe (III) -carboxylate complexes.

### 3.8. Kinetic study

The kinetics of the Fenton reaction may be very complicated, because a large number of intermediates are generated during the reaction and a large number of steps are involved, so these steps are indistinguishable from a macroperspective [44]. Therefore, in this research, the kinetic of the Fenton reactions with zero-, first-, and second-order kinetics for all concentrations of ARG, according to Eqs. (1)-(3), in different current densities or pH have been investigated.

(1)C0-Ct=k0t

(2)ln (C0/Ct)=k1t

(3)(1/C0)-(1/Ct)=k2t

**Figure 8:**
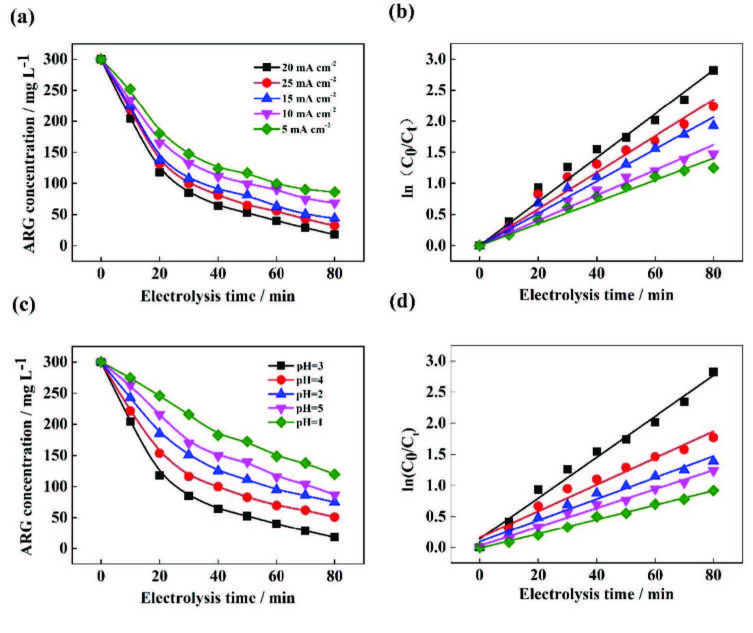
Concentration changes of ARG and pseudo-first-order reaction kinetics at ((a), (b)) different current densities and ((c), (d)) different pH.

Where C_0_ and C_t_ are the concentrations of ARG before and after treatment, Figure 8a and 8c show the concentration changes of ARG under different current densities and different pH conditions. k_0_, k_1_, and k_2_ represent the kinetic constants of the degradation of ARG, respectively, t is the reaction time.The linear regression analysis is shown in Table 3, the average value of R^2^ of first-order kinetics is larger; therefore, the pseudofirst-order model is the best model to describe the decolorization of ARG at different current density or pH using the Fenton process. As mentioned above, the current density and pH affect the concentration of Fe^2+^ and H_2_O_2_ in the solution, which in turn affects the progress of the Fenton reaction and the •OH. As can be seen from Figures 8b and 8c, the change in trend of rate constant (k) and •OH production is the same, this is because •OH oxidation is the most important reason for ARG degradation (reaction (9) and (10)), the amount of •OH produced determines the rate of degradation. Under the condition of high •OH production, the reaction rate is bound to be higher, while under the condition of low •OH production, the reaction rate must also be reduced.

**Table 3:** Results of the kinetics of EF-Feox process for different concentrations of ARG.

k0(mg L-1 min-1)		Pseudo-zero-order		Pseudo-first-order		Pseudo-second-order	
		R2	k1×10-3(min–1)	R2	k2×10-3(mg L-1 min−1)	R2	
Current density (mA cm–2)	510152025	2.562.642.873.072.98	0.85860.84730.82280.80450.8205	16.3418.5023.8733.0226.68	0.97310.97960.98490.98360.9740	0.10.10.20.60.3	0.990.99450.97540.8530.9448
pH	12345	2.282.653.072.792.6	0.97850.89370.80450.84130.9394	11.6317.2133.0221.4015.26	0.99450.97520.98360.96530.9877	0.060.10.60.20.1	0.98120.9960.8530.98970.9781
R2 average	0.8611			0.9802		0.9556	

## 4. Conclusions

In this paper,EF-Feox process was used to remove ARG with graphite felt as the cathode and an iron plate as the anode. Among them, Fe^2+^ was generated by anodic oxidation of iron plate at the surface of the electrode, and H_2_O_2_ was generated by cathodic reduction of O_2_. When the reaction device was turned on, two-electron-based reactions occurred at the anode and cathode at the same time, theoretically generating Fe^2+^ and H_2_O_2_ with the same number of moles, promoting the effect of the Fenton reaction. The main results of the experiments could be summarized as follows: when the electrolyte Na_2_SO_4_ concentration was 0.2 M, the plate spacing was 2 cm, the initial concentration of ARG was 300 mg/L, the reaction time was 80 min, the pH was 3, and the current density was 20 mA/cm^2^, the removal effect of ARG was the best, reaching 94.05%. On the other hand, when pH = 3, the current density = 15 mA/cm^2^, H_2_O_2_, Fe^2+^ and •OH have the best formation rate. Moreover, it was concluded that the removal of ARG occurs with first-order reaction kinetics.
